# Disentangling specific and unspecific components of innate immune memory in a copepod–tapeworm system

**DOI:** 10.3389/fimmu.2024.1307477

**Published:** 2024-01-29

**Authors:** Tze Hann Ng, Mark C. Harrison, Jörn P. Scharsack, Joachim Kurtz

**Affiliations:** Institute for Evolution and Biodiversity, University of Münster, Münster, Germany

**Keywords:** immune priming, innate immune specificity, *Macrocyclops albidus*, *Schistocephalus solidus*, tapeworm-copepod system

## Abstract

Evidence that the innate immune system can respond with forms of memory upon reinfection has been accumulating over the past few years. These phenomena of “immune priming” in invertebrates, and “trained immunity” in vertebrates, are contrary to previous belief that immune memory and specificity are restricted to the adaptive immune system. However, while trained immunity is usually a response with rather low specificity, immune priming has shown highly specific responses in certain species. To date, it is largely unknown how specificity in innate immune memory can be achieved in response to different parasite types. Here, we revisited a system where an exceptionally high degree of innate immune specificity had been demonstrated for the first time, consisting of the copepod *Macrocyclops albidus* and its natural parasite, the tapeworm *Schistocephalus solidus*. Using homologous (same family) vs. heterologous (different family) priming-challenge experiments, we first confirm that copepods exposed to the same parasite family benefit from reduced secondary infections. We further focused on exposed-but-not-infected copepods in primary exposure to employ a transcriptomic approach, distinguishing between immunity that was either specific or unspecific regarding the discrimination between tapeworm types. A weighted gene co−expression network (WGCN) revealed differences between specific and unspecific immunity; while both involved histone modification regulation, specific immunity involved gene-splicing factors, whereas unspecific immunity was primarily involved in metabolic shift. We found a functional enrichment in spliceosome in specific immunity, whereas oxidative phosphorylation and carbon metabolism were enriched in unspecific immunity. Our findings allow discrimination of specific and unspecific components of an innate immune memory, based on gene expression networks, and deepen our understanding of basic aspects of immune systems.

## 1 Introduction

Immunological memory was long believed to be a characteristic exclusively present in adaptive immune systems. In vertebrates, antigen-specific adaptive immunity is driven by T cells, B cells, and dendritic cells and has a long-lasting immunological memory (1, 2). However, it has been questioned whether the adaptive immune system is the only one capable of immunological memory (3–5). There is growing evidence that innate immune systems, in both humans and other vertebrates, enable enhanced responses after reinfection (6, 7). This response in the vertebrate immune system is known as innate immune memory or “trained immunity,” which is also relevant for non-specific protection against COVID-19 (8–10). In recent studies, live Bacillus Calmette–Guérin (BCG) vaccines protected against unrelated diseases by trained immunity triggering non-specific protection (9, 11). Trained immunity may not be antigen specific but rather mediated via epigenetic reprogramming, metabolic and/or functional alterations showing broad protection (12).

Invertebrates and plants were both reported to have forms of immune memory (13, 14). Systemic acquired resistance (SAR) is a reaction that confers immunological memory in plants by altering the host defense mechanisms epigenetically (14). In invertebrates, line-specific memory was first reported in a copepod–tapeworm system, in *Macrocyclops albidus*, by repeated exposures to related *Schistocephalus solidus* tapeworms versus unrelated tapeworm families (15). Among insects, *Bombus terrestris* and *Tribolium castaneum* show specific protection in response to homologous (i.e., same species) bacterial exposure (16, 17). The model organism *Drosophila melanogaster* shows immune priming (18) that may include tolerance rather than resistance (19). Moreover, a mosquito–*Plasmodium* system shows hemocyte differentiation for non-specific memory induced by gut microbiota during penetration of *Plasmodium* ookinetes across the gut barrier (20).

Evidence of immune priming has now been reported in arthropods, ctenophores, mollusks, cnidarians, and nematodes (21, 22). Priming varies in specificity, i.e., the ability to distinguish between parasites. However, the molecular basis of such specificity in immune priming is unclear. Recent transcriptome analyses suggest possible mechanisms involved in immune priming. For example, phagocytes are involved in priming in *D. melanogaster* (18); snail *Biomphalaria glabrata* innate immune memory reveals a shift from a cellular immune response (encapsulation) to a humoral immune response (biophalysin) (23) and regulates a diverse set of pattern recognition molecules and effector repertoires in response to different strains of *Schistosoma* parasites (24); priming of *T. castaneum* induces expression of a diverse set of immune genes upon challenge with the same bacteria (25); and the involvement of immune genes and lysosomes during priming using gut bacterial symbionts has been shown in *Anopheles gambiae* (26). Increased immune priming specificity with different gene expression profiles related to immune, metabolic and transcription-modifying genes can rapidly evolve in *T. castaneum* through experimental selection (27). In another study of the freshwater platyhelminth *Schmidtea mediterranea*, it was demonstrated that *Staphylococcus aureus*–primed worms induced epigenetic reprogramming involving peptidoglycan receptor and histone methyltransferase genes, which in turn enhanced bacterial clearance much earlier upon second infection (28). Immune priming in invertebrates partially resembled trained immunity that undergoes metabolic and epigenetic changes, indicating that mechanisms for innate immune memory are to some extent evolutionarily conserved (28–30).

Such accumulating evidence has led to considerable recent debates as to what is needed to define immunological memory. While some authors focus on the aspect of specificity to define memory (4), others have put more emphasis on the aspect of extinction, i.e., that the original immune activation vanishes before re-exposure (31). Pradeu and Du Pasquier (32) make the important point that “the most fruitful way of dealing with immunological memory nowadays was to adopt a multidimensional and gradual conception of immunological memory.” They consider the five key dimensions strength, speed, extinction, duration, and specificity. Realizations of memory in the animal kingdom may fulfill these five criteria more or less clearly. We here focus on the aspect of specificity, because the copepod–tapeworm system used in the present study was the first to show that highly specific innate immune memory is present in invertebrates, using homologous and heterologous challenges (15). Here, we hypothesized that the specificity of innate immune memory is mediated by molecular mechanisms that are distinguishable from more general, unspecific induced immune responses. We thus followed a similar experimental design and additionally employed a transcriptomic approach to infer the molecular basis of the induced immune priming with specificity against antigenic characteristics of this parasite. For a combination of homologous versus heterologous priming-challenge experiments, adult male copepods were primed with tapeworm larvae, followed by a second exposure to parasites derived from either the same tapeworm family or a different family. Immunological specificity was determined by the infection status after primary and secondary exposure. Consequently, fluorescently labeled tapeworms were used for secondary exposure to facilitate distinguishing them from primary exposure. The molecular basis induced by immune priming was tested by weighted gene co−expression network analysis of the transcriptomic data. Our approach enabled us to decompose immune priming reactions into specific and unspecific components, each of which involved different gene regulatory networks.

## 2 Materials and methods

### 2.1 Experimental copepods

Experimental copepods (*M. albidus*) were initiated with 32 mated female copepods randomly collected from laboratory cultures, originally collected in Northern Germany, as described in (33). Cultures were maintained under laboratory conditions at 20°C with a 16:8 light:dark cycle, as described in (34).

### 2.2 Experimental parasites

The tapeworm *S. solidus* has a complex life cycle, where cyclopoid copepods and three-spined stickleback fish serve as intermediate hosts, while warm-blooded vertebrates, mostly fish-eating birds, are suitable definitive hosts (35). *S. solidus* can be kept in the laboratory, where the definitive host is replaced by *in vitro* breeding, thereby enabling experimental infections of the intermediate hosts (36–38). The *S. solidus* larvae used here were F_1_ offspring of wild-caught parasites from three-spined sticklebacks collected in April and October 2019 from the brook Ibbenbürener Aa (Germany, 52°17′33.51′′N, 7°36′45.46′′E). The tapeworms were bred *in vitro* in size-matched pairs by outcrossing. Briefly, *in vitro* breeding was done as described (36, 37), and parasite eggs were stored in sterilized tap water at 4°C in darkness. For experiments, parasite eggs were incubated for 3 weeks at 20°C in the dark and subsequently exposed to light to induce hatching.

### 2.3 Labeling of parasites

Fluorescent tracer dye CMAC (Molecular Probes) was used to label *S. solidus* coracidia (parasite larvae), as described previously (39). Briefly, coracidia were immersed in 20 µM CMAC in 2 mL sterile tap water at 20°C for 1 h in the dark. Before infection, 8 mL sterile tap water was added to dilute surplus dye.

### 2.4 Experimental infection

The infection protocol was modified from a previous report (34). Laboratory-bred adult male copepods were individualized in wells of 24-well plates with 2 mL tap water and exposed to one hatched live *S. solidus* larva. Seven *S. solidus* larva families were used for infection of copepods. The *S. solidus* larvae from the same two parasite parents are here referred to as a family. All copepods were fasted for 2 days before infection and fed three artemia every other day after infection.

### 2.5 Experimental design

**Figure 1:**
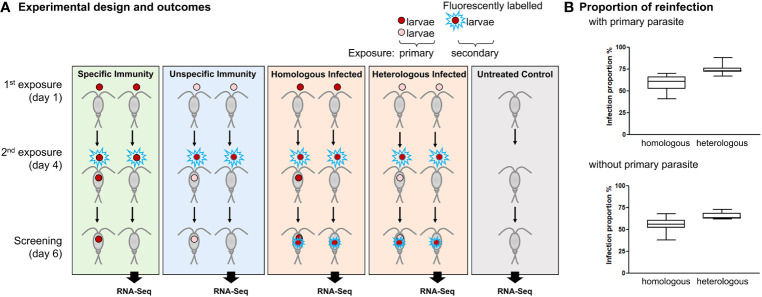
Homologous exposure using larvae from the same parasite family reduced reinfection compared to heterologous exposure using larvae from different parasite families. **(A)** Experimental design and outcomes. As an example, two parasite families are shown (red and pink coracidia, respectively), whereas eight such combinations were used in the experiment. Copepods were exposed to one parasite coracidium and subsequently exposed to the same or a different parasite family at 4 days post-primary exposure. All coracidia used for secondary exposure were fluorescently labeled to distinguish them from the coracidia used for primary exposure. The reinfection proportion was recorded at 6 days post-primary exposure. According to the resulting infections, copepod samples were categorized into the following outcome groups: Specific Immunity (i.e., copepods resisting homologous reinfection), Unspecific Immunity (i.e., copepods resisting heterologous reinfection), Homologous Infected and Heterologous Infected (i.e., copepods that did not resist reinfection), and Untreated Control (i.e., sham exposed). Only copepods that cleared the primary infection were used for a transcriptomic analysis (shown as RNA-Seq) at 6 days post-primary exposure, to examine gene regulations in each of these groups. **(B)** Proportion of copepod reinfections between homologous exposure (left box) or heterologous exposure (right box). N = 7 parasite families for experiments. Means and standard errors are indicated.

The experimental design closely followed a previous study (15) and added a follow-up host transcriptomic analysis (see **Figure 1** for an overview of the experimental design). In the present study, each individual copepod was exposed to one *S. solidus* parasite larva (called coracidium at this stage) for primary exposure and, 4 days later, to another coracidium for secondary exposure. For the secondary exposure, either a coracidium derived from the same parasite sibship (i.e., full-sib family) as the one that had been used for primary exposure was used (the treatment was denoted “homologous”), or a coracidium derived from a different family was used (“heterologous”); see **Figure 1**. To create these combinations, we made use of seven parasite families that had been experimentally bred in the laboratory from worm pairs (i.e., outcrossed, full-sib families; see Section 2.2). For primary and secondary exposure, we combined coracidia derived from these families to form eight combinations of homologous or heterologous exposures (as we had only seven parasite families, and one parasite family was used twice; see **Table 1** for details). Fluorescently labeled parasites were used exclusively for the secondary exposure, which allowed us to discriminate labeled parasites resulting from the secondary exposure from the unlabeled ones resulting from primary exposure. For this, copepods were screened alive, using bright-field and epifluorescence microscopy (absorption/emission of 354 nm/466 nm), at 6 days post-primary exposure. At this age, both labeled and unlabeled parasites (which are called plerocercoids at this stage) are easy to spot inside the copepod. Copepods were sacrificed after screening and stored in RNA later for subsequent RNA sequencing (see Section 2.6). Although we did not screen the copepods after each parasite exposure (to minimize stress), we did check for the presence of coracidium in the 24-well plates after each exposure and the absence of coracidium after 1 h of exposure, which confirms that the copepods had ingested the coracidium.

**Table 1:** Proportion of infections with or without a primary parasite in secondary exposure.

					Proportion of infections in secondary exposure	
Combination	Parasite family	Experimental group	Treatment	Number of copepods	With a primary parasite	Without a primary parasite
1	IBB1	IBB1xIBB1	Homologous	160	0.41	0.56
		IBB4xIBB1	Heterologous	131	0.76	0.62
	IBB4	IBB4xIBB4	Homologous	150	0.67	0.56
		IBB1xIBB4	Heterologous	145	0.67	0.63
2	IBB17	IBB17xIBB17	Homologous	100	0.54	0.38
		IBB18xIBB17	Heterologous	93	0.72	0.62
	IBB18	IBB18xIBB18	Homologous	94	0.57	0.59
		IBB17xIBB18	Heterologous	96	0.73	0.67
3	IBB7	IBB7xIBB7	Homologous	259	0.65	0.68
		IBB8xIBB7	Heterologous	249	0.72	0.70
	IBB8	IBB8xIBB8	Homologous	252	0.70	0.62
		IBB7xIBB8	Heterologous	263	0.76	0.73
4	IBB18	IBB18xIBB18	Homologous	44	0.51	0.48
		IBB20xIBB18	Heterologous	43	0.73	0.63
	IBB20	IBB20xIBB20	Homologous	40	0.65	0.56
		IBB18xIBB20	Heterologous	39	0.88	0.64

To determine the proportion of infections resulting from primary and secondary exposure, the number of copepods with unlabeled parasites (primary infection) and with labeled parasites (secondary infection) were recorded for each experimental group. The proportion was determined by dividing the number of infected copepods by the number of exposed copepods in each experimental group (**Table 1**; **Supplementary Table 1**).

For transcriptomic analyses, to minimize the potential effects of a resident primary infection in our samples, only copepods exposed but not infected in primary exposure were included. The time point 6 days post-primary exposure (i.e., 2 days post-secondary exposure) was chosen because we were interested in differences in transcriptomic responses to the secondary exposure regarding successful clearance of either homologous or heterologous primary exposure, taking into account that it needs some time for the parasite to enter the body cavity. The copepod samples were categorized into the following outcome groups (**Figure 1**): Specific Immunity (homologous exposed but not infected); Unspecific Immunity (heterologous exposed but not infected), Homologous Infected (homologous exposed and infected), Heterologous Infected (heterologous exposed and infected), and Untreated Control (sham exposed). The categories “Specific Immunity” and “Unspecific Immunity” are expected to differ regarding the specificity of the immunity that results from the cleared primary exposure and is relevant to protect the host against secondary exposure: specific immunity protects against reinfection with the same parasite family, whereas unspecific immunity cross-protects against secondary infection with a different parasite family.

### 2.6 Sample preparation and library construction and sequencing

RNA sequencing was conducted using samples from two biological experiments with two parasite families each (family numbers 7, 8 and 17, 18, respectively). Only copepods that had cleared the primary infection were used for RNA sequencing. Each outcome group consisted of three replicates with three individual copepods in each replicate. Thereby, there were 12 samples for Specific Immunity, 12 samples for Unspecific Immunity, 12 samples for Homologous Infected, 12 samples for Heterologous Infected, and 6 samples for Untreated Control. In total, 54 RNA samples were extracted with RNeasy Plus Micro Kit (Qiagen), following the manufacturer’s instructions. RNA libraries were created following polyA selection using the NEBNext Ultra II Directional RNA Library Prep kit and sequenced on two lanes of the NextSeq 500 system with 2 × 150 bp paired reads per sample at the Core Facility Genomics of the Medical Faculty, University of Muenster, Germany. The number of raw reads is shown in **Supplementary Table 2**.

### 2.7 Quality control and *de novo* assembly

A *de novo* transcriptome was assembled with Trinity (40, 41), enabling determination of gene expression profiles in non-model organisms without a sequenced genome. Briefly, raw read quality was first checked with FastQC (42) and trimmed using Trimmomatic 0.36 (43) to remove low-quality bases, adapter contamination, and reads shorter than 50 bp. STAR (44) was used to map reads to the *S. solidus* reference genome downloaded from WormBase ParaSite (45). Mapped reads were filtered, with remaining unmapped reads referred to as clean reads. The number of parasite reads and clean reads is shown in **Supplementary Table 2**. Note that parasite reads were also mapped in samples of exposed-but-not-infected copepods. Alive young parasite larvae are easily detected with fluorescent dye labeling; however, dead larvae are undetectable. In exposed-but-not-infected copepods, there might thus be RNA from freshly dead larvae. While most parasites are eliminated early in the infection, i.e., in the gut or during gut wall passage (34), some parasites may be killed later, producing relatively high parasite read numbers. To avoid uncertainty, we removed five samples from the groups due to high numbers of mapped reads in those samples.

For *de novo* assembly, only samples from exposed-but-not-infected and sham-exposed groups were used to form a draft transcriptome of *M. albidus* using Trinity software version 2.11.0, followed by assembly quality assessment with BUSCO (46) using the Arthropoda dataset (arthropoda_odb10) in transcriptome mode. To reduce assembly redundancy and duplicated genes, all transcripts were clustered using CD-HIT-EST (47) and quality was reassessed with BUSCO. Next, clustered transcripts were identified for candidate protein-coding regions based on open reading frame (ORF) prediction by TransDecoder v5.5.0 (41). Potential coding transcript sequences were annotated using a Trinotate (48) pipeline by Trinity against the sequence database (BLAST+/Swiss-Prot), protein domain identification (HMMER/PFAM), protein signal peptide and transmembrane domain prediction (signalP/tmHMM), and eggNOG/Gene Ontology (GO)/Kyoto Encyclopedia of Genes and Genomes (KEGG) databases.

To estimate transcript abundance in a genome-free manner, Salmon (49) was used to construct the reference transcriptome index using the draft transcriptome, and the abundance of the clean paired-end reads of each sample was estimated. Next, matrices of counts and expression values were constructed. The matrix of expression values before cross-sample normalization (TPM) were used for downstream analyses of the gene co−expression network (GCN).

### 2.8 Construction of the weighted gene co−expression network

To identify co-expressed gene modules within outcome groups, WGCNA (50) uses correlation to identify sets of genes (eigengenes) that are expressed together and the values for each eigengene can be used in a similar was as the original gene expression values. We used eigengene data to identify differentially expressed modules between each outcome group versus Untreated Control. All analyses of GCN were performed with R v4.1.1 (51) and RStudio v1.4.1717 (52). Before running WGCNA packages, the TPM matrices were filtered with a cutoff of total counts of at least 10 in 2 samples, and then DESeq2 package (53) was used to normalize and transform the matrices. Next, batch variation was removed with the removeBatchEffect function from the limma package (54) to maintain the reliability of the network construction results. Co-expression networks were constructed independently for each outcome group, which included 15–18 samples each, employing the filtered matrices of 16,312 genes. Networks were constructed with soft threshold power = 14, signed networks, Pearson correlation, maxBlockSize = 20,000, and minModuleSize = 30. Network preservation statistics (55) were calculated at the module level across each network using the built-in WGCNA function, *modulePreservation* and the *Zsummary* statistic, and scored at least 8, indicating moderate to strong evidence of module.

### 2.9 Identification of the module of interest and functional enrichment

Modules with differential expression across the outcome group and Untreated Control were discerned using empirical Bayes statistics with the limma package in R. The top significant upregulated module for each group was used to construct KEGG BRITE functional hierarchies (56) and heatmap plots. The same identified modules were then subjected to the Search Tool for the Retrieval of Interacting Genes (STRING) database v11.5 (57) to construct protein–protein interaction (PPI) gene networks and examined for functional enrichment. Unfortunately, most *M. albidus* sequences mapped against the copepod (*Tigriopus californicus*) database were so far annotated as domain-containing protein or uncharacterized proteins; thus, the fly (*D. melanogaster*) database was mapped to improve functional annotations.

### 2.10 Identification of differential transcript usage

Differential transcript usage (DTU) via SuperTranscripts (58) was conducted by utility in the Trinity toolkit. SuperTranscripts built a supertranscriptome that provided a genome-like reference for studying the gene with differential transcript usage (i.e., differential exon usage). Briefly, Corset (58) was used to generate clusters of the transcripts as inputs to Lace (58) to construct a supertranscriptome for each cluster. The supertranscriptome of each cluster was then assigned to a gene and used to map the raw reads through STAR (44). Feature-Counts (59) from the Subreads R package was used to count mapped reads to the “exonic” regions and to generate a count matrix. This count matrix was then subsequently used for DTU expression analysis using DEXSeq (60). A default threshold was used to extract the significant transcript regions and identify the top 50 genes that were subjected to PANTHER (61) for protein class analysis.

## 3 Results

### 3.1 Immunological specificity in copepod defense reduces risk of reinfection

To test for specificity in innate immune memory, individual *M. albidus* copepods were exposed to one *S. solidus* tapeworm larva and, at 4 days post-primary exposure, were re-exposed to a fluorescently labeled tapeworm larva derived from either the same or a different parasite family (**Figure 1A**). Individual infection success was evaluated in a total of 2,158 copepods (**Table 1**). Prior exposure to the same parasite family (homologous exposure) resulted in less secondary reinfection compared to exposure to a different parasite family (heterologous exposure) (**Figure 1B**, **Table 1**). The average reinfection success was reduced from 75% ± 6% to 59% ± 10% in primary infected copepods (paired Student’s *t*-test, t = 3.989, p = 0.0026). Likewise, in copepods without primary parasite, reinfection was reduced from 65% ± 4% to 55% ± 9% (t = 4.243, p = 0.0019) (**Figure 1B**). This confirms the previously demonstrated immunological specificity in this host–parasite system (15).

### 3.2 Gene regulation profiles associated with immunological specificity

To examine gene regulation, we performed RNA sequencing of copepod hosts from a subset of the whole experiment, focusing on two independent biological experiments where copepods had been exposed to two parasite families each, i.e., four parasite families in total. Further, only exposed-but-not-infected copepods in primary exposure were included for RNA sequencing (**Figure 1A**), thereby avoiding potential confounding effects of resident primary parasites. Copepods that eliminated the primary parasite can be considered showing an immune priming effect; if there is a specific memory inherent in the defense, we would expect a different gene regulation in the outcome group Specific Immunity (homologous exposed but not infected) compared to Unspecific Immunity (heterologous exposed but not infected). We further included Homologous Infected (homologous exposed and infected) and Heterologous Infected (heterologous exposed and infected) to detect differences between non-infected and infected copepods and an Untreated Control (sham-exposed, non-exposed copepods). Each of these four experimental groups consisted of six replicates (containing three individual copepods each) per experiment, plus three Untreated Controls per experiment, i.e., 54 RNA libraries in total.

**Figure 2:**
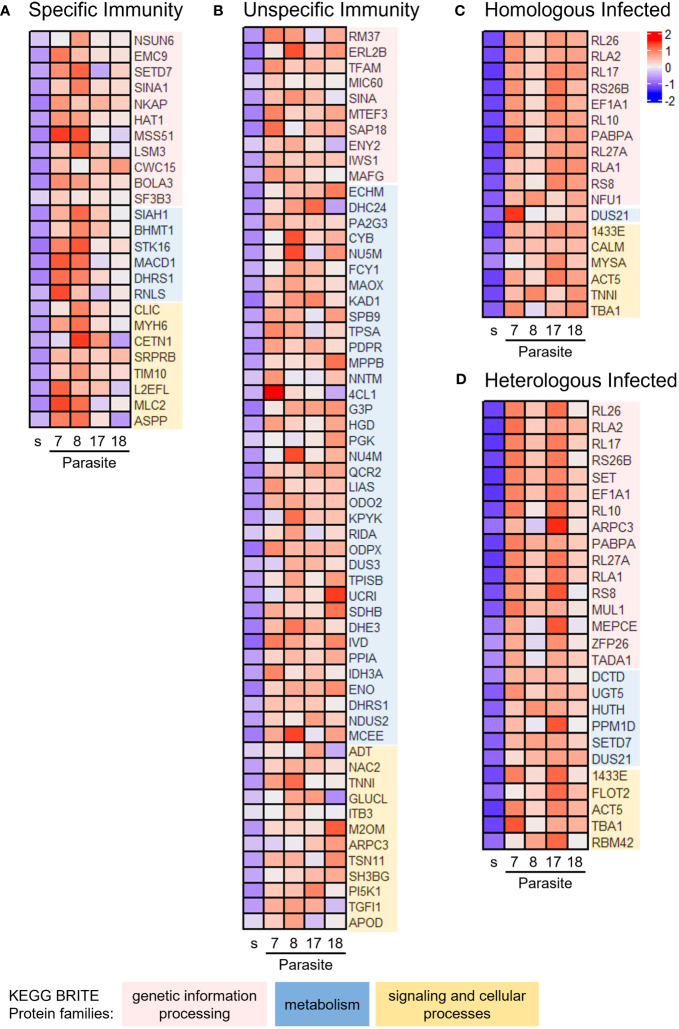
Immunological specificity–associated genes enriched in copepods from homologous versus heterologous exposure. Co-expression modules were constructed for each outcome group using weighted gene co-expression network analysis (WGCNA). Differentially expressed modules were identified in each outcome group versus Untreated Control, based on the overall expression of eigengene values in the module. The top differentially upregulated modules were subjected to BRITE functional hierarchies. Heatmap of eigengene expression in **(A)** Specific Immunity, **(B)** Unspecific Immunity, **(C)** Homologous Infected, and **(D)** Homologous Infected. See **Supplementary Table 3** for further information on gene annotation. s = Untreated Control. Parasite families denoted 7, 8, 17, and 18.

To obtain a holistic understanding of the immunological specificity response, we used co-expression network analysis, considering transcripts across copepods exposed to the four parasite families in each outcome group Specific Immunity, Unspecific Immunity, Homologous Infected, and Heterologous Infected. We constructed four weighted co-expression networks and identified differentially upregulated modules using the empirical Bayes statistics by comparing each outcome group versus Untreated Control. We selected the top differentially upregulated module from each outcome group for KEGG BRITE functional hierarchy analysis (**Figure 2**; **Supplementary Table 3**). All modules were mapped to genetic information processing, metabolism, and signaling and cellular processes. Specific Immunity was associated with more upregulated genes of genetic information processing (**Figure 2A**), whereas Unspecific Immunity was associated with more upregulated genes of metabolism (**Figure 2B**). Similar to Specific Immunity, most genes mapped to genetic information processing in Homologous Infected and Heterologous Infected (**Figures 2C, D**). Additionally, we observed that all outcome groups have genes involved in epigenetic control of transcription (**Supplementary Table 3**). Histone acetyltransferase (HAT) and RNA transcription, translation, and transport factor protein (RTRAF) were found in the Specific Immunity module, while histone deacetylase (HDAC) was found in the Unspecific Immunity module. In contrast, there were histone acetylation inhibitors (e.g., template-activating factor-i) and transcriptional repressors (e.g., heterochromatin protein 1) in Homologous Infected and Heterologous Infected modules. Of note, cytoskeleton proteins were found in all outcome groups, indicating that parasite invasion had a significant impact on the cytoskeleton, such as disruption of the gut wall during parasite migration from gut to hemocoel.

**Figure 3:**
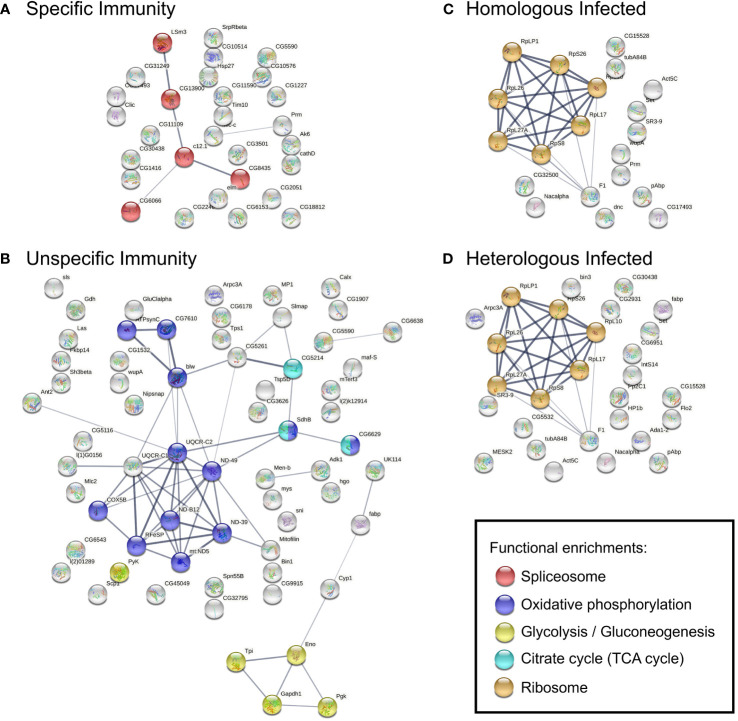
PPI networks constructed using genes in selected modules, **(A)** Specific Immunity, **(B)** Unspecific Immunity, **(C)** Homologous Infected, and **(D)** Homologous Infected. Construction based on the *D. melanogaster* database. Nodes represent proteins and contain the protein known or predicted 3D structure, colored accordingly to KEGG functional enrichments in the network. Edges represent protein–protein interactions; line thickness indicates the experimentally determined strength of interactions based on the STRING database. FDR < 0.05 for functional enrichments in networks.

We next questioned the extent to which the enriched pathway and protein–protein interaction network influenced immunological specificity response profiles. The genes in the top differentially upregulated module of each network (**Supplementary Table 3**) were then used for pathway enrichment and protein–protein interaction analysis. Specific Immunity revealed genes mapped to the spliceosome (**Figure 3A**), such as U6 small nuclear RNA (snRNA)–associated Sm-like protein LSm3, protein CWC15 homolog, and NKAP family protein (**Supplementary Table 4**). Unspecific Immunity was enriched in oxidative phosphorylation and carbon metabolism [glycolysis, tricarboxylic acid (TCA) cycle] (**Figure 3B**; **Supplementary Table 4**). Several enzymes such as ATP synthase, reduced nicotinamide adenine dinucleotide (NADH) dehydrogenase, succinate dehydrogenase, and cytochrome c oxidase/reductase are involved in oxidative phosphorylation; while glyceraldehyde 3-phosphate dehydrogenase, phosphoglycerate kinase, and pyruvate kinase are involved in glycolysis. In contrast, Homologous Infected and Heterologous Infected modules were functionally coherent, with the majority of genes mapped to the ribosome pathway (**Figures 3C, D**; **Supplementary Table 4**).

### 3.3 Differential transcript usage analysis across specific immunity versus unspecific immunity

As noted in **Figure 3A**, the spliceosome pathway was enriched in Specific Immunity. We next attempted to use differential transcript usage analysis to look for potential changes caused by messenger RNA (mRNA) splicing at the transcript level. We compared Specific and Unspecific Immunity to identify genes with differential transcript usage (gDTU) in response to Specific Immunity. The gDTU lists from each comparison were examined further for various protein classes (**Supplementary Table 5**). Considering the measurement variations of RNA sequencing (RNA-Seq) experiments, we focused only on gDTUs replicated in at least two independent datasets of parasite families to select genes consistently altered in Specific Immunity (**Table 2**). Four protein classes were identified, namely, chaperones (e.g., heat shock proteins), cytoskeleton proteins, metabolite interconversion enzymes, and transporters.

**Table 2:** Selected genes with DTU (gDTU) associated with Specific Immunity compared to Unspecific Immunity.

Protein class	Gene symbol	Gene name (orthology)	Parasite family			
			7	8	17	18
Chaperone (PC00072)	Hsc70-4	Heat shock 70 kDa protein cognate 4			X	X
	Hsp83	Heat shock protein 83		X	X	
Cytoskeletal protein (PC00085)	Act57B	Actin-57B			X	X
	Mhc	Myosin heavy chain, muscle	X	X		
	Act5C	Actin-5C		X	X	
Metabolite interconversion enzyme (PC00262)	Argk	Arginine kinase	X	X		
	Pxd	Peroxidase		X	X	X
Transporter (PC00227)	ATPsynbeta	ATP synthase subunit beta			X	X

## 4 Discussion

While the existence of memory in innate immune systems has now been demonstrated in both vertebrate and invertebrate animals, the degree of specificity and its mechanistic basis are largely unclear and may vary across taxa. We here used a naturally evolved system consisting of a copepod host and its tapeworm parasite, where a high degree of specificity within innate immune memory had previously been demonstrated for the first time (15). Our present study provides an important confirmation of this experiment, as it revealed consistent results to demonstrate specific memory in this system. On average, after repeated parasite exposure, reinfection success was reduced by ~10% in infected copepods for homologous exposure, i.e., repeated exposure to the same type of parasite, here using sibling parasites that are assumed to show antigenic similarity, compared to heterologous exposure to different parasite families (**Figure 1**). Likewise, a reduction in reinfection success by approximately 10% was also observed in (15) for outcrossed parasites (as were used in the present study), while the effect size doubled for antigenically even more similar parasites resulting from single selfing in this hermaphroditic tapeworm. There were similar results in copepods without a resident primary parasite, indicating that the effect was not due to, e.g., parasite competition within host, but rather resulting from a specific defense after consecutive exposure to related parasites. While the higher clearance following homologous exposure indicates immune specificity, heterologous exposed-but-not-infected copepods may also show immune activation compared to previously non-exposed hosts, i.e., a primed but unspecific reaction. We thus denoted this group “Unspecific Immunity” to compare its gene expression with homologous exposed hosts (denoted “Specific Immunity”).

To shed light on the underlying mechanisms of specificity in this host–parasite system, we studied whole-transcriptome gene expression, focusing on hosts that were able to clear both infections (on day 1 and day 4), thereby avoiding potentially confounding effects of resident parasites. Our whole-body transcriptome analyses examined the extent to which gene expression differed between homologous and heterologous exposed copepods under repeated challenges. Selection for immunological specificity in antigenic resemblance resulted in different transcriptional responses during secondary exposure (**Figures 2**, **3**), indicating potential regulation of immunological specificity and the degree of plasticity of this invertebrate’s defense. According to the current basic model for innate immune memory in invertebrates, priming to a primary infection can cause a cellular response that can either be sustained for a long time in a way that protects against a secondary infection (sustained response) or be recalled in a way that is stronger and faster after a secondary infection (recalled response), or it can cause a shift to a humoral response upon secondary infection (62). The innate immune memory, trained immunity, provides enhanced responses to subsequent triggers but is the result of long-term immune memory adaptation without specificity. It involves epigenetic modulation and metabolic reprogramming (63). However, priming can be specific or unspecific. Identifying mechanisms for the differential specificity of invertebrate immunity is challenging, given the invertebrate system’s ability to perform multiple functions in parallel. In the present study, we observed that primed copepods showed transcriptional patterns associated with epigenetic modulation during secondary infection. We did not find any strong signals of classical immune pathways or immune receptors that could mediate specificity. These could either not be involved in defense against tapeworms at the studied time point, or they were too diluted in the whole-body transcriptomes. However, focusing on certain tissues would be challenging in these tiny copepods, where we lack knowledge about immune-relevant organs.

In line with the transcriptomic patterns pointing toward a role of epigenetic processes, pathogen priming and transgenerational immune priming (TGIP) involve epigenetic changes in insects and brine shrimp (27, 64–67), with transcriptional reprogramming of HAT and HDAC playing a role in TGIP, as shown in *Manduca sexta* (65). Histone acetylation and deacetylation are associated with gene transcription and memory formation. Histone acetylation by HATs promotes active transcription, whereas HDACs repress it (68). Accordingly, our findings in Specific and Unspecific Immunity versus Homologous Infected and Heterologous Infected show transcriptomic patterns indicating epigenetic changes via histone acetylation (**Figure 2**; **Supplementary Table 3**). HAT upregulation was observed in Specific Immunity, but HDAC expression in Unspecific Immunity and histone acetylation inhibitor in Homologous Infected and Heterologous Infected. This may suggest increased active transcription of genes in response to specific priming. Despite a lack of information regarding histone modification in gene regulations in copepods, studies on other crustaceans, like *Daphnia* and *Artemia*, have been described (69, 70). Additionally, a study in copepods observed a reactivation of transcription processes from diapause-to-post-diapause transition by upregulating genes involved in histone acetylation and downregulating genes associated with chromatin silencing at 1 h post-collection. This was followed by the sequential and sustained upregulation of several genes during the 14-day experimental period (71). We thus assume that the observed transcriptomic patterns associated with histone modifiers are indicative of conserved epigenetic processes.

Histone acetylation has been shown to influence alternative splicing via a kinetic competition between transcription elongation and splicing. During transcription, the dynamic cycle of histone acetylation and deacetylation modulates the elongation rate and splicing pattern (72–74). Nucleosomes that are hyperacetylated by HATs enhance the rate of Pol II elongation when pre-mRNA is transcribed, and may result in increased gene expression and exon skipping (75, 76). Although most studies of histone modification and alternative splicing have been conducted in mammals and *Drosophila*, we found that genes that are upregulated in the Specific Immunity condition are involved in histone acetylation (i.e., HAT), modulation of mRNA transcription by Pol II (i.e., RTRAF) and the spliceosome (**Figures 2**, **3**), indicating the possibility of chromatin structure and splicing pattern modulation during transcription in these copepods. Interestingly, in neuron cells, external signals caused histone acetylation and rapid RNA polymerase II to affect the isoform selection of neural cell adhesion molecule (ncam) gene (77). In arthropods, there is another immunoglobulin (Ig) superfamily of adhesion molecules called Down syndrome cell adhesion molecule (Dscam) gene (78). Dscam isoforms can be produced by RNA splicing and were previously shown to be involved in immunity and have the ability to discriminate different pathogens to some extent (79–84); thereafter, Dscam was suggested to be a potential pattern recognition receptor (PRR) that is involved in immune priming of arthropods (21, 22, 85). Unfortunately, Dscam was not detected in either co-expression network or transcript usage analysis. We used total RNA isolated from the whole copepod body; future study using specific immune tissue may investigate the potential involvement of Dscam-specific immunological memory further.

In our study, the general immune/stress responders heat shock proteins and peroxidase were genes for which we could identify different transcript usage in Specific versus Unspecific Immunity (**Table 2**; **Supplementary Table 5**). When organisms are exposed to heat stress or pathogens, they overexpress heat shock proteins (Hsps). Hsps are a large family of chaperones with many isoforms (86, 87); recent advances in RNA sequencing revealed at least 90 Hsp90 isoforms produced by alternative splicing in wheat (88). Hsps are not only chaperone proteins in adaptive immunity; some chaperones have peptide binding sites as well, albeit with relatively low specificity (89). In *Artemia* exposed to heat shock, an acquired trait of high Hsp70 levels and pathogen resistance was associated with histone acetylation and DNA methylation (70). We identified differential transcript usage in genes from both main classes of heat shock proteins, Hsp70 and Hsp90 (i.e., *hsp83* gene). Hsp90 is a chaperone involved in developmental processes and supposed to be an evolutionary capacitor (90). Its expression has been shown to be related to immunity in the insect *T. castaneum* (91), but a potential role of alternative splicing for priming has not been tested so far.

Furthermore, double peroxidase and HAT are required for hemocyte differentiation factor synthesis in the *Plasmodium–*mosquito system in order to maintain the non-specific immune memory response (92). Another antioxidant system component, superoxide dismutase (SOD), has been shown to be involved in *T. castaneum* immune priming (93). Peroxidases are a diverse enzyme family with at least 15 members. In vertebrates, plants, and bacteria, peroxidase isoforms can be produced via alternative splicing (94), but information on crustaceans is still lacking. In our study, we speculate that the specific protective response in invertebrates is influenced by changes in histone modification, splicing patterns, and stress response factors. However, further study at earlier time points may be needed to better understand the fate and potential of PRRs and effectors during the specific immune response in this copepod–tapeworm system, as specific recognition and effector functions may not be fulfilled by the same molecules (95, 96).

Taken together, our gene expression data suggest that immune priming in *M. albidus* may partially resemble trained immunity that is based on epigenetic processes. Such memory could be specific by regulating histone modification on gene splicing. Indeed, our findings support the view that the immune system exhibits tremendous evolutionary flexibility across taxa. Immune memory in innate and adaptive immunity has been proposed to form an evolutionary continuum in which a more robust immune response first evolved via epigenetic processes, and specificity later developed in a subgroup of animals (vertebrates) via gene recombination (2, 97). In addition to this model, we propose that specificity may develop in another subgroup of animals, i.e., crustaceans, through transcript splicing.

## Data availability statement

The sequencing results for this study can be found in National Center for Biotechnology Information’s (NCBI) Sequence Read Archive, https://www.ncbi.nlm.nih.gov/sra (BioProject: PRJNA1023184).

## Ethics statement

Animal experimental procedures were executed in accordance with EU Directive 2010/63/EU for animal experiments, and all applicable international, national, and/or institutional guidelines for the use of animals in experiments were followed. Sticklebacks were maintained and treated with approval of the local veterinary and animal welfare authorities under Project Number 84-02.04.2014.A368.

## Author contributions

TN: Conceptualization, Formal analysis, Methodology, Writing – original draft, Writing – review & editing. MH: Formal analysis, Writing – review & editing. JS: Methodology, Writing – review & editing. JK: Conceptualization, Methodology, Supervision, Writing – original draft, Writing – review & editing.
